# A Myb transcription factor, *Pg*Myb308-like, enhances the level of shikimate, aromatic amino acids, and lignins, but represses the synthesis of flavonoids and hydrolyzable tannins, in pomegranate (*Punica granatum* L.)

**DOI:** 10.1093/hr/uhac008

**Published:** 2022-02-11

**Authors:** Rohit Dhakarey, Uri Yaritz, Li Tian, Rachel Amir

**Affiliations:** Department of Plant Science, Migal – Galilee Technology Center, P.O. Box 831, Kiryat Shmona 1101600, Israel; Department of Plant Science, Migal – Galilee Technology Center, P.O. Box 831, Kiryat Shmona 1101600, Israel; Department of Biotechnology, Tel-Hai College, Upper Galilee 1220800, Israel; Department of Plant Sciences, University of California, Davis, CA 95616, USA; Department of Plant Science, Migal – Galilee Technology Center, P.O. Box 831, Kiryat Shmona 1101600, Israel; Department of Biotechnology, Tel-Hai College, Upper Galilee 1220800, Israel

## Abstract

Pomegranate fruit peels are highly abundant in metabolites derived from the shikimate pathway, such as hydrolyzable tannins (HTs) and flavonoids. These metabolites are beneficial to human health (commercial juice is enriched with peel metabolites), and also protect the fruit from environmental stresses. To understand the transcriptional control of shikimate pathway-related metabolites in pomegranate, we cloned and characterized a subgroup S4 R2R3 Myb transcription factor, *Pg*Myb308-like. Overexpressing *PgMyb308-like* in pomegranate hairy roots increased the accumulation of shikimate, aromatic amino acids, isoferulic acid, and total lignins, but led to reduced gallic acid and its downstream products HTs, as well as multiple flavonoids. Changes in these metabolites are supported by the increased expression of *3-deoxy-D-arabino-heptulosonate 7-phosphate synthase* and *shikimate dehydrogenase 1* (*PgSDH1*) (the *SDH* isoform associated with shikimate biosynthesis), and the reduced expression of *PgSDH4* (the *SDH* isoform suggested to produce gallic acid). Transcriptome analysis of *PgMyb308-like-*overexpressing hairy roots further revealed reprogramming of cell wall-related genes, while overexpression of *PgMyb308-like* in *Arabidopsis thaliana* plants uncovered its distinct role in a different genetic and metabolic background. These results together suggest that *Pg*Myb308-like activates genes in the shikimate pathway and lignin biosynthesis, but suppresses those involved in the production of HTs and flavonoids.

## Introduction

Pomegranate fruits are well known for their beneficial properties towards human health. Consumption of pomegranate juice (PJ) has been associated with reduction in the occurrence and symptoms of stress-related chronic inflammatory diseases and age-related sicknesses, such as cardiovascular diseases [1], carcinogenesis [2], neurodegeneration, obesity, and diabetes [3].

**Figure 1:**
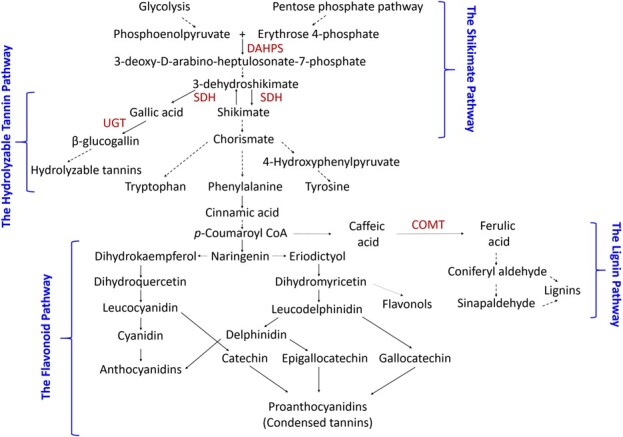
The shikimate pathway delivers precursors for aromatic amino acid (AAA), flavonoid, lignin, and hydrolyzable tannin (HT) biosyntheses. Dashed arrows represent multiple biosynthetic steps. Selected enzymes in the shikimate, lignin, and HT pathways are shown. SDH, shikimate dehydrogenase; DAHPS, 3-deoxy-D-arabino heptulosonate-7-phosphate synthase; UGT, UDP-dependent glycosyltransferase; COMT, caffeic acid 3-*O*-methyltransferase.

The health beneficial properties of pomegranate products have been attributed to the high level of phenolic compounds that are found in fruit peels. A major group of these phenolics are hydrolyzable tannins (HTs), particularly the two isomers (and) of punicalagins that are the most abundant HTs in pomegranate fruit peels [4]. Punicalagins accumulating in pomegranate fruit peels are also responsible for more than 50% of the juice’s potent antioxidant activity, because HTs from fruit peels are carried over to juice due to the commercial whole fruit extraction method [5]. In addition to HTs, the outer peels (~1 mm from the surface of the peel) of fruits also contain another group of phenolic compounds, anthocyanins, which not only provide numerous health benefits, but also contribute to the red-magenta color, a desirable commercial trait for attracting consumers to purchase the fruit [6]. Besides HTs and anthocyanins, the outer peels are also enriched in a wide variety of flavonoids, *e.g.* flavonols, flavones, proanthocyanidins, flavanones, and isoflavonoids, as well as additional plant specialized metabolites, *e.g.* benzoate derivatives, alkaloids, quinones, suberin, and lignins [7]. Because most of these metabolites are derived from the shikimate pathway or its downstream products the aromatic amino acids (AAAs) [reviewed by [8]] (Fig. 1), the outer peels of pomegranate fruit would be an ideal tissue to investigate the intricate regulatory control of these diverse groups of shikimate-associated metabolites.

Regulation of these shikimate-associated metabolites is essential because of their critical roles in plant growth, development, and interactions with the environment. Since the shikimate pathway provides precursors for their biosynthesis (Fig. 1), it is perceivable that precise spatiotemporal coordination of their metabolism is needed throughout development, and in response to different environmental conditions. Indeed, studies have shown that synthesis of the shikimate pathway and its associated metabolites is regulated by transcription factors (TFs) belonging to several different families, such as Myb, bHLH, WD-40, and WRKY TFs [8]. In addition, it was found that several TFs not only regulate genes in the shikimate pathway but also control genes in pathways that are downstream of the shikimate pathway or the AAAs [e.g. [9–11]], suggesting the presence of coordinated gene expression networks connecting core/primary and specialized/secondary metabolism in plants [12]. A few notable examples include the petunia (*Petunia hybrida*) ODORANT1, an R2R3-type Myb TF that controls genes in the shikimate and phenylpropanoid pathways [13], as well as the Myb30, Myb55, and Myb110 TFs in rice that induce the expression of genes in the shikimate pathway together with those in the cinnamate/monolignol pathway [14]. Moreover, several TFs act as activators to genes in certain shikimate related pathways, but repressors of genes in other shikimate related pathways, often in response to developmental cues or environmental conditions [e.g. [15–17]].

Of all the Myb TFs identified in plants, R2R3 Myb TFs constitute the largest group and play important roles in multiple biological processes, encompassing core and specialized metabolic reactions [18]. The R2R3 Myb TFs contain the conserved Myb domain in their N-terminal region for DNA binding, and the modulator domain in their C-terminal region for the regulatory activity [15]. According to the motifs present in the C-terminal region, R2R3 Myb TFs can be further classified into 28 subgroups; genes from several subgroups were found to be related to the shikimate pathway and its associated metabolites [15]. However, despite the emerging roles of TFs in controlling the production of shikimate and its associated metabolites in other plant species, their function in pomegranate is largely unknown. In this study, we identified an R2R3 Myb TF, *Pg*Myb308-like, expressing in the outer peels of pomegranate fruits that contain a high level of anthocyanins, HTs, and other phenolic compounds [6, 19–21]. *PgMyb308-like* was overexpressed in pomegranate hairy roots and *Arabidopsis thaliana* plants to assess their functions *in planta*. Comparative metabolite and gene expression analysis of the overexpression and the empty vector (EV) control pomegranate hairy root and *A. thaliana* lines revealed its function in regulating the expression of genes in the shikimate and phenolic metabolic pathways as well as its broader impact on cell wall reprogramming.

## Results and discussion

### Protein sequence and phylogenetic analyses of *Pg*Myb308-like suggest that it belongs to the subgroup S4 of R2R3 Myb TFs

**Figure 2:**
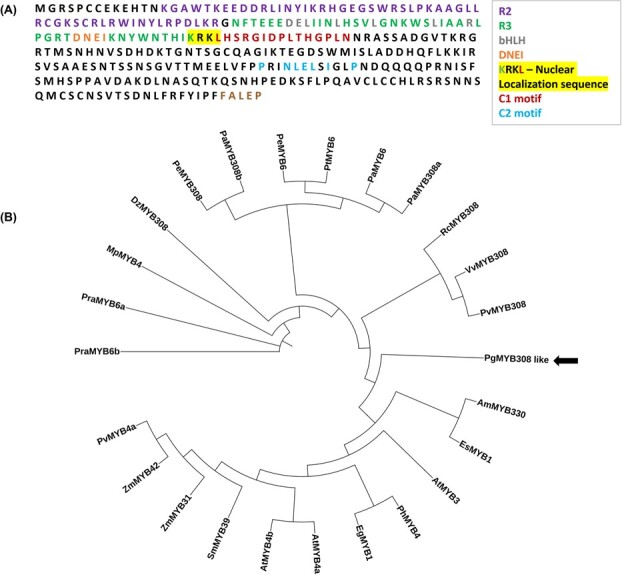
Sequence analysis of *Pg*Myb308-like. (A) The amino acid sequence with the conserved motifs. The R2 and R3 domains are indicated by violet and green color. The bHLH-binding domain (within the R3 domain) and the DNEI domain are indicated in gray and orange. The C1 and C2 motifs are indicated in red and blue. The last five amino acids (marked in brown) at the C-terminus differ from the sequence previously deposited in the NCBI (accession number: AIO09658.1). (B) A cladogram of *Pg*Myb308-like and additional R2R3-Myb transcription factors that have similar motives from other plants. The accession numbers are listed in Supplementary Fig. S2.

To isolate R2R3 Myb TFs from the outer peels of pomegranate, we designed the forward primer according to the 5′-end sequence of the conserved R2 motif and the reverse primer that binds to the poly-T tail of the cDNA. Several R2R3 Myb genes were amplified from a cDNA library constructed from the outer fruit peels of the ripening “Wonderful” accession using this set of primers. One of the R2R3 Mybs was identical to a TF previously deposited in GenBank (accession number: AIO09658.1)*, except for* the last five amino acids (Fig. 2A). To verify if this gene involves regulating the shikimate pathway, the expression level of this gene was measured in the outer peels of two pomegranate accessions with pink and red peel color 21, in three developmental stages: young (stage 3), nearly mature fruit (stage 8), and ripened fruit (stage 10) as characterized previously [22]. Its transcripts decreased during fruit development, similar to the reduced content of gallic acid, ellagic acid, and catechin in both accessions and decreased punicalagins in the pink peel accession (Supplementary Fig. S1). These results suggest that this Myb TF may regulate the production and/or accumulation metabolites in the shikimate and its related pathway, and therefore was selected for further analysis. Sequence analysis indicated that the protein contains 309 amino acids with a predicted molecular weight of 34.4 KDa. *In silico* analysis (http://nls-mapper.iab.keio.ac.jp/cgi-bin/NLSMapper _form.cgi) revealed the presence of a putative nuclear localization signal that positions at amino acids 113–116 (Fig. 2A).

The newly identified R2R3 Myb is homologous to a Myb308-like TF from *Ricinus communis (RcMyb308-like)* and a Myb308-like TF from *Populus alba* with *57.24%* and *53.73% sequence identity, respectively, and was therefore designated Pg*Myb308-like. By comparing the sequence of *Pg*Myb308-like to those of other R2R3 Myb TFs, four additional motifs besides the conserved R2R3 motifs were identified (Fig. 2A; Supplementary Fig. S2). These include: (i) an N-terminal [D/E]Lx2[K/R]x 3Lx6Lx3R domain, which is the interacting motif with bHLH co-factors [17]; (ii) a DNEI motif, which is a conserved element immediately adjacent to the bHLH binding region that has been associated with the regulation of proanthocyanidin biosynthesis [23]. This motif has been detected in members of subgroups S4 and S5 of R2R3 Myb TFs [23]; (iii) a C1-like motif that usually contains the sequence of LlsrGIDPxT/SHRxI/L [24]. However, *Pg*Myb308-like contains a slightly varied sequence of LhsrGIDPlT/HgpL (amino acids that differ from the conserved motif are underlined). The C1-like motif was found in Myb repressors of flavonoid biosynthesis [25], while was also suggested to act as an activator in other pathways [24, 25]; and (iv) a C2-like motif that typically has the sequence of pdLNLD/ELxiG/SxP, though *Pg*Myb308-like contains the sequence of prINLELsiGlP (i.e. the amino acids dL were replaced with rI in *Pg*Myb308-like*)*. This is a signature motif for subgroup S4 R2R3 Myb TFs, which reportedly function as repressors for the synthesis of phenylpropanoid-derived compounds [24, 25].

Consistent with the presence of a C2-like motif, the phylogenetic analysis also showed that *Pg*MYB308-like clustered with two subgroup S4 R2R3 Mybs: Myb330 of *Antirrhinum majus* and Myb1 of *Epimedium sagittatum* (*Es*Myb1) (Fig. 2B). *Es*Myb1 was proposed to act as a transcriptional repressor of the phenylpropanoid pathway and the flavonoid pathway in *E. sagittatum* [26]. Together, the phylogenetic analysis and the classification of R2R3 Mybs [15] suggest that *Pg*MYB308-like belongs to subgroup S4 TFs that include Myb 308, Myb 330, Myb 1, Myb 12, Myb 11, Myb 4, Myb 6, Myb 8, and Myb 32 TFs, with demonstrated regulatory roles in the metabolism of monolignols, proanthocyanins, anthocyanins, flavonols, and sinapate esters [15].

**Figure 3:**
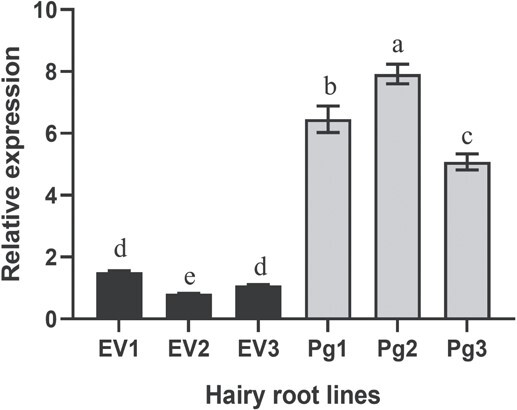
Expression analysis of transgenic pomegranate hairy roots. The gene expression data were normalized with the pomegranate *Ribosomal Protein S* gene. Pg1-Pg3, hairy root lines overexpressing *PgMyb308-like*. EV1-EV3, empty vector control lines. Each hairy root line was derived from an independent transformation event. The data are means of three biological replicates with error bars indicating SD. Different letters indicate significantly different (*P* < 0.01) expression, based on the Tukey–Kramer test.

### Overexpression of *PgMYB308-like* in pomegranate hairy roots led to increased shikimate, AAAs, and lignins, and reduced HTs and flavonoids

To determine the *in planta* function of *Pg*Myb308-like, the full-length *PgMyb308-like* cDNA was cloned into a Gateway binary vector harboring a *green fluorescent protein* (*GFP*) marker gene, and the constitutive cauliflower mosaic virus (CaMV) *35S* promoter to drive the expression of *PgMyb308-like*. The empty vector (EV) without *PgMyb308-like* was used as a negative control. These plasmids were transformed into *Agrobacterium rhizogenes*, which were then used to induce transgenic pomegranate hairy roots, an established experimental system [27] that produces a high level of HTs as found in fruit peels [28]. Among the eight hairy root lines that were transformed with *PgMyb308-like* and showed green fluorescence under the microscope (indicating the expression of *GFP*), three lines, Pg1, Pg2, and Pg3, were selected for further analysis because of their high expression levels of *PgMyb308-like* relative to those in the three EV control lines (Fig. 3).

To understand the function of *Pg*Myb308-like in controlling metabolites synthesized from the shikimate and downstream pathways, gas chromatography–mass spectrometry (GC–MS) analysis was carried out to examine the level of shikimate and AAAs in the hairy roots expressing an EV or overexpressing *PgMyb308-like*. For this analysis and other metabolic analyses, the EV controls (EV1, EV2, and EV3), or the *PgMyb308-like* overexpression lines (Pg1, Pg2, and Pg3) were grouped together and analyzed as a set.

**Figure 4:**
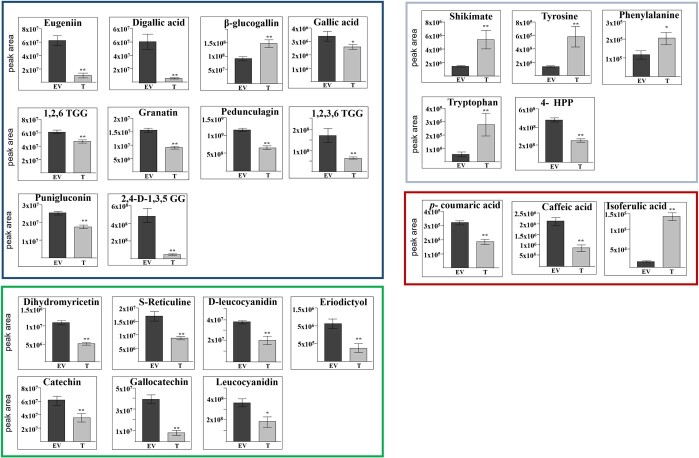
The contents of metabolites related to the pathway of shikimate, phenylpropanoid, and hydrolyzable tannin in transgenic hairy roots overexpressing *PgMyb308-like* (T) or an empty vector (EV). Metabolites related to the synthesis of shikimate and aromatic amino acids are in a gray frame; hydrolyzable tannins are in a blue frame; flavonoids are in a green frame; and hydroxycinnamic acids in a red frame. The Y-axis represents the normalized peak area. The results are average ± SE of three biological repeats of transgenic lines and each biological repeat has three technical repeats. Statistical significance is based on Student’s *t*-test (*P* < 0.01, and *P* < 0.001) and marked by one and two asterisks, respectively. 1,2,6-TGG, 1,2,6-Trigalloylglucose; 1,2,3,6-TGG, Tetra-*O*-galloyl-β-D-glucose; 2,4-digalloyl-1,3,5-GG, 2,4-*O*-digalloyl-1,3,6-tri-*O*-beta-D-galloylglucose; 4-HPP, 4-Hydroxyphenylpyruvate.

The level of shikimate was increased by 4.8-fold in hairy roots overexpressing *PgMyb308-like* as compared to EV lines (Fig. 4). Of the AAAs, tyrosine was increased by 5.5-fold, tryptophan by 5-fold, and phenylalanine by 1.8-fold (Fig. 4). It was previously suggested that in the outer pomegranate peels, shikimate and gallic acid biosyntheses compete for the common precursor 3-dehydroshikimate [20]; the shikimate dehydrogenase (SDH)-catalyzed reaction can produce gallic acid or shikimate depending on the cell’s redox state [29]. Shikimate subsequently leads to the biosynthesis of AAAs, whereas gallic acid is the precursor for HT biosynthesis [29, 30]. Interestingly, in contrast to increased shikimate and AAAs, gallic acid was reduced by 26% in hairy roots overexpressing *PgMyb308-like* relative to EV controls (Fig. 4).

In addition to the GC–MS analysis, liquid chromatography–tandem mass spectrometry (LC–MS/MS) analysis was also performed to quantify HTs and other specialized metabolites related to the shikimate pathway, such as flavonoids, in the transgenic hairy roots. Forty-one annotated metabolites were detected, thirty-one of which were significantly changed in hairy roots overexpressing *PgMyb308-like* compared with EV (Supplementary Table S1). Principal component analysis (PCA) indicated that the first principal component (PC1) has a strong positive correlation with the lines overexpressing *PgMyb308-like* and a strong negative correlation with the EV lines (Supplementary Fig. S3).

The LC–MS/MS results also showed that among the 21 detected HTs, β-glucogallin, the first product of the HT pathway, was increased by 52% in *PgMyb308-like*-overexpressing lines (Fig. 4; Supplementary Table S1). However, several other HTs were significantly reduced compared with EVs. These include digallic acid reduced by 12-fold, 2,4-*O*-digalloyl-1,3,6-tri-*O*-beta-D-galloylglucose by 10.4-fold, eugeniin by 6.5-fold, 1,2,3,6-tetra-*O*-galloyl-β-glucose by 2.7-fold, granatin by 2.2-fold, pedunculagin by 76%, punigluconin by 40%, and casuarinin by 58% (Fig. 4). Ellagic acid and brevifolin carboxylic acid also had a trend of reduction by 44% and 23%, but were not statistically significant (Supplementary Table S1). Those HTs that did not differ between *PgMyb308-like*-overexpressing and EV lines include the punicalagin isomers, 1,2,3,4,6-pentagalloyl glucose, castalagin, 3,4,3′-tri-*O*-methylellagic acid, punicalin, valoneic acid dilactone, 1,2,6-trigalloyl-β-D-glucose, and lagerstannin C (Supplementary Table S1).

In contrast to the increased tyrosine, its intermediate metabolite, 4-hydroxyphenylpyruvate (Fig. 1), was reduced by 87% (Fig. 4; Supplementary Table S1). In addition, several flavonoids, which are derivatives of phenylalanine, accumulated at much lower levels in hairy roots overexpressing *PgMyb308-like* compared to EVs. These include gallocatechin that was reduced by 4.7-fold, eriodictyol by 2.7-fold, dihydromyricetin by 2.1-fold, *S*-reticuline by 92%, leucocyanidin by 89%, deoxyleucocyanidin by 82%, catechin by 73%, epigallocatechin by 58%, and dihydroquercetin by 14% (Fig. 4; Supplementary Table S1). The LC–MS/MS analysis also detected three hydroxycinnamic acids: caffeic acid, *p*-coumaric acid, and isoferulic acid (Fig. 4). Caffeic acid, a precursor for the synthesis of lignin monomers [31], was decreased by almost 87%. Notably, while caffeic acid was reduced, the level of isoferulic acid, an isomer of ferulic acid and likely a downstream product of caffeic acid, was increased by 10.4-fold (Fig. 4).

Like caffeic acid, ferulic acid also functions as a building block for the synthesis of lignin monomers [32]. Additionally, ferulic acid plays an important role in crosslinking polysaccharides (such as arabinoxylans), and coupling lignins and proteins to strengthen the cell wall [reviewed by [31]]. Although ferulic acid was not detected in the transgenic hairy roots, the significantly changed caffeic acid and isoferulic acid content in *PgMyb308-like*-overexpressing lines prompted us to assess total lignins in the transgenic hairy roots. The total lignin content in hairy roots overexpressing *PgMyb308-like* was significantly elevated by 70% relative to EV [119 ± 31 and 70 ± 19 mg/g dry weight (DW), respectively]. These findings are in accordance with the report for *EsMyb1*, another *R2R3 Myb* in subgroup S4. While the preferential expression of *EsMyb1* in lignin-rich tissues of *E. sagittatum* implicates its possible role in regulating lignin biosynthesis, *EsMyb1* was suggested to act as a transcriptional repressor of the phenylpropanoid and the flavonoid pathway genes [33]. In addition, when the loblolly pine *PtMyb8* (a member of the S4 subgroup) was overexpressed in white spruce, genes associated with shikimate and monolignol biosyntheses were upregulated, contrasting the down-regulated flavonoid pathway genes [9]. However, overexpression of *AmMyb308* and *AmMyb330*, two *A. majus* Myb TFs in the S4 subgroup, in tobacco suppressed the expression of hydroxycinnamic acid biosynthetic genes and led to reduced lignins, while having moderate effects on flavonoid production [34]. The results from the current study and previous reports suggest that Myb308 and several other subgroup S4 Mybs [15] play a role in modulating lignin biosynthesis, which can occur through up- or down-regulation depending on the Myb TFs and the plant species/tissue types.

Overall, the metabolite analysis indicated that gallic acid as well as multiple HTs, flavonoids, and hydroxycinnamic acids were significantly reduced in the *PgMyb308-like*-overexpressing hairy roots. In contrast, shikimate, AAAs, isoferulic acid, and total lignins showed increased accumulation when *PgMyb308-like* was overexpressed. These results suggest that *Pg*Myb308-like may activate the expression of shikimate pathway genes and those involved in isoferulic acid and lignin biosynthesis. At the same time, it acts as a repressor for genes related to the HT, flavonoid, and hydroxycinnamic acid biosynthetic pathways. *Pg*Myb308-like can regulate more than one class of phenylpropanoid-derived compounds (flavonoids and hydroxycinnamic acids), similar to other S4 Myb TFs, such as *Md*Myb3 in apple that regulates anthocyanins and flavonols, *At*Myb4 in *A. thaliana* that regulates monolignols and phenolic acids, and *At*Myb75 (PAP1) in *A. thaliana* [9] that regulates monolignols, anthocyanins, proanthocyanidins, flavonols, and phenolic acids [reviewed by [15]]. It is possible that different TF partners or cofactors (co-activators or co-repressors) may be recruited to act together with *Pg*Myb308-like to regulate different pathways through activation or repression. It is also possible that the histone modification and chromatin remodeling machinery may play a role in the differential pathway regulations (activation *vs* repression) by *Pg*Myb308-like.

**Figure 5:**
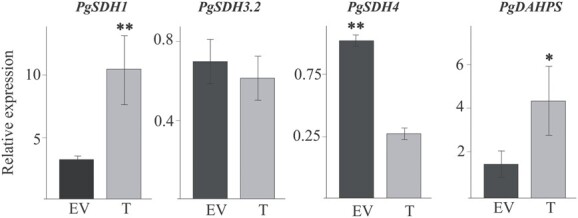
Quantitative real-time PCR analysis of genes related to the shikimate pathway. Expression of *3-deoxy-D-arabino-heptulosonate-7-phosphate synthase* (*PgDAHP*S) as well as *shikimate dehydrogenase 1* (*PgSDH1*), *PgSDH3.2*, and *PgSDH4* is shown*.* The expression levels were normalized with pomegranate *Ribosomal Protein S.* The results are average ± SE of three biological replicates of transgenic lines, each with three technical replicates. Statistical significance was determined based on Student’s *t*-test (*P* < 0.05 and *P* < 0.01) and marked with one and two asterisks, respectively.

### Expression of *PgSDH1*, *PgSDH4*, and *PgDAHPS* was altered in transgenic hairy root lines overexpressing *PgMyb308-like*

The contrasting changes in shikimate and gallic acid levels in hairy roots overexpressing *PgMyb308-like* suggests a possible change in the expression of *PgSDHs* that position at the branching point between shikimate and gallic acid biosyntheses [29]. Shikimate is produced from 3-dehydroshikimate using NADPH as a cofactor, whereas gallic acid is formed in the presence of NADP^+^ [29]. A previous study showed that *Vitis vinifera* contains four isozymes of *Vv*SDH (*Vv*SDH1-*Vv*SDH4), which have different affinities to NADPH and NADP^+^ [30]. The study in *V. vinifera* also suggested that HT biosynthesis depends mainly on the activity of *Vv*SDH3 and *Vv*SDH4, while *Vv*SDH1 plays a major role in controlling shikimate production [30]. Similarly, of the six *PgSDHs* identified in pomegranate, the expression of *PgSDH3.2* and *PgSDH4* previously showed a positive correlation with HT levels in a fruit peel culture system [20], suggesting that they might control the production of gallic acid and thus also HTs. To investigate the potential regulation of *PgSDHs* by *Pg*Myb308-like, the expression of *PgSDH1, PgSDH3.2,* and *PgSDH4* in the transgenic hairy roots was analyzed using quantitative real-time PCR. *PgSDH4* expression was reduced by 2.5-fold, *PgSDH1* expression was increased by 3.4-fold, and *PgSDH3.2* expression was not significantly changed in hairy roots overexpressing *PgMyb308-like* compared to EV (Fig. 5). The expression of *PgDAHPS* that encodes the first enzyme in the shikimate pathway was also determined by quantitative real-time PCR and showed a 2.8-fold increase in hairy roots overexpressing *PgMyb308-like*. These results suggest that *Pg*Myb308-like activates *PgDAHPS* and *PgSDH1* in the shikimate pathway, supporting the overall increased shikimate and AAA accumulation in these hairy roots, while represses *PgSDH4* that leads to the synthesis of gallic acid (Fig. 4). The non-significantly changed *PgSDH3.2* expression also suggests that *Pg*SDH4 could be the major *Pg*SDH that contributes to gallic acid and HT production in hairy roots.

### Transcriptome analysis of pomegranate hairy roots overexpressing *PgMyb308-like* uncovered major changes in expression of cell wall-related genes

To understand more broadly how *Pg*Myb308-like may regulate genes in different metabolic pathways, transcriptome analysis was performed on hairy roots overexpressing *PgMyb308-like* and EV control using the TranSeq method [35]*.* About 87% of the sequencing reads were mapped to the pomegranate genome and ~ 94% of the uniquely mapped reads were found in exons. Of the 18 831 genes identified, 267 were differentially expressed genes (DEGs) (|log_2_Fold Change| > 1, adjusted *P* < 0.05) in *PgMyb308*-like-overexpressing lines vs. EV controls, which include 194 and 72 genes that were up-regulated and down-regulated, respectively.

Gene ontology (GO) analysis was carried out to determine the biological functions of DEGs in the transgenic hairy roots. Out of the 267 DEGs, 207 were successfully mapped to GO-SLIM (Supplementary Fig. S4). GO enrichment analysis was also conducted and the enrichments of particular cellular components (CC) and biological function (BP) were determined using the PANTHER Overrepresentation test (Supplementary Table S2). This analysis compares the frequency of the total number of genes annotated to the particular CC or BP, to the frequency of the number of genes in the input list falling under the same GO term. The PANTHER Overrepresentation test of the DEGs showed a significant (*P* < 0.001) positive fold enrichment of CC related to cell wall (5.07), cell periphery (2.44), extracellular matrix (26.19), extracellular region (2.33), external encapsulating structure (5.56), and secretory vesicle (6.82) (the fold enrichment is shown in parenthesis; Supplementary Table S2A). All of these enriched GO terms are associated with cell wall modulation, including the secretory vesicle category that plays a significant role in the synthesis of cell wall and its components. Together, the GO CC enrichment analysis corroborates the changes detected in the lignin content and supports the hypothesis that *Pg*Myb308-like regulates the programming of cell wall.

The GO BP analysis showed that genes related to cellulose metabolism processes were significantly (*P* < 0.001) increased by 13.5 to 14.8-fold. In addition, other genes related to phenylpropanoid and secondary metabolites, ion transport, and defense to biotic stresses were also significantly (*P* < 0.001) increased (Supplementary Table S2B). This analysis suggest a link between lignin and the carbohydrate components in the cell wall [36], and cell wall modulation. It is also connect between the biotic stresses defense to the cell wall and the phenolic metabolites, since they are both rapidly remodeled in response to attack by fungi and bacteria [37].

Among the 194 up-regulated genes, 34 are homologous to genes that were not previously characterized. Within the 160 genes with annotated functions, 33 were related to cell wall, 43 were related to abiotic and biotic stresses, 12 were involved in metal chelation (mainly Fe), and 4 were classified in the group of riboflavin (vitamin B2) biosynthesis (Supplementary Table S3). The other annotated genes were not grouped into clusters (Supplementary Table S3). Genes fell into the cell wall category can be further classified into three groups (Supplementary Table S4). DEGs in the first group (7 genes) are related to the synthesis of monolignols and lignins. They include: (i) caffeic acid 3-*O*-methyltransferase (COMT), a key enzyme involved in lignin biosynthesis and the formation of ferulic esters in the cell wall (Fig. 1); (ii) uclacyanin-3-like that is suggested to acts as an electron carrier involved in lignin formation; (iii) laccase 7-like and laccase 13 that play a role in lignin formation by promoting the oxidative coupling of monolignols; (iv) peroxidase 72-like and peroxidase 10 that are able to oxidize different substrates using hydrogen peroxide as electron donor, and play a significant role in lignin biosynthesis by cross-linking its monomers; and (v) phenylcoumaran benzylic ether reductase that exists mainly in lignifying cells and was suggested to be associated with the lignification process. The up-regulation of these 7 genes are likely related to the higher levels of isoferulic acid and total lignins found in hairy roots overexpressing *PgMyb308-like*.

DEGs in the second group (8 genes) are associated with the cell wall structure. These include: (i) expansin-A18 and extensin-2-like, which lead to extension and loosening of plant cell walls by distracting non-covalent bonding between matrix glucans and cellulose microfibrils; (ii) glycine-rich cell wall structural protein 1.8 that acts as scaffolds or agglutinating agents for the deposition of cell wall constituents; (iii) two of glycine-rich cell wall structural protein-like that suggested to function in cell wall maintenance and repair during dehydration and rehydration; (iv) WAT1-related protein that is required for secondary cell wall deposition; (v) probable xyloglucan endotransglucosylase/hydrolase, which is involved in the modification of cell wall components; (vi) pectinesterase 3-like, which is a abundant cell wall-associated enzyme that enables plant cell wall modification and subsequent breakdown; and (vii) cuticle protein 64-like that is involved in cuticle synthesis of the epidermis. It was interesting to observe that overexpression of *PgMyb308-like* in hairy roots led to additional changes in genes related cell wall structure in addition to those related to lignin metabolism.

DEGs in the third group (4 genes) are those related to modifications of carbohydrate polymers in the cell wall. These include: (i) endoglucanase 5 that is involved in cell wall modification, cellulose and other polysaccharide degradation; (ii) lichenase-like that solubilizes and degrades β-glucans in plant cell walls; (iii) glucan endo-1,3-$ \square$ $-glucosidase 14-like that is involved in cell wall biogenesis/degradation; and (iv) probable xyloglucan endotransglucosylase/hydrolase that modifies cell wall components. Lignins form complex with carbohydrates such as cellulose and hemicellulose [36]. Overexpression of *PgMyb308-like* in hairy roots appeared to orchestra a concerted change in the structure of and various polymers in the cell wall.

Taken together, the results from the GO CC enrichment and the DEG expression analyses indicated that *Pg*Myb308-like regulate a large number of cell wall-related genes, which likely lead to modification of cell wall components and cell wall structure. Genes related to the degradation of polysaccharides such as cellulose, β-glucans, and xyloglucan, and those related to lignin synthesis were up-regulated. In particular, the enhanced expression of *COMT* may explain high levels of isoferulic acid and total lignin detected in *PgMyb308-like*-overexpressing hairy roots.

In addition to the genes involved in the cell wall structure, genes that are related to phenolic metabolism were also up-regulated. These include: (i) polyphenol oxidase, chloroplastic-like that catalyzes the oxidation of *O*-diphenols to produce *O*-quinones; (ii) 1,4-dihydroxy-2-naphthoyl-CoA synthase that is involved in the synthesis of 1,4-dihydroxy-2-naphthoyl-CoA, a derivative of chorismate, and takes part in quinol/quinone metabolism; (iii) 4-hydroxyphenylpyruvate dioxygenase that is central for tyrosine and phenylalanine catabolism, and the biosynthesis of plastoquinone and tocopherols in plants; (iv) xanthotoxin 5-hydroxylase CYP82C2-like that functions in the furanocoumarin biosynthetic pathway; and (v) berberine bridge enzyme-like 18 that catalyzes the stereospecific transformation of the *N*-methyl group of (*S*)-reticuline into the C-8 berberine bridge carbon of (*S*)-scoulerine in isoquinoline alkaloid metabolism (Supplementary Table S3).

Among the 72 genes that were down-regulated in hairy roots overexpressing *PgMyb308-like*, 15 are homologous to genes that have not been functionally characterized in plants (Supplementary Table S5). From the 57 annotated genes, 6 are involved in responses to biotic and abiotic stresses, and 25 are involved in the metabolism of shikimate and its related metabolites. Within the last group, 20 genes are related to cell wall structure and composition and can be divided into two subgroups (Supplementary Table S6). The first subgroup (6 genes) includes genes related to lignins and lignans: (i) peroxidase 24-like and peroxidase 5-like that are involved in the biosynthesis and degradation of lignins; (ii) dirigent protein 21-like, which binds free radical monolignol species and coordinates their coupling during lignin biosynthesis. It also yields active lignans from two molecules of coniferyl alcohol in the biosynthesis of lignans and flavonolignans; (iii) glucuronoxylan 4-*O*-methyltransferase 1, which takes part in hemicellulose synthesis. Reduction in its activity was also correlated with altered lignin composition [38]; (iv) chitinase-like protein 2 that is required for proper cell wall biosynthesis and preventing lignin accumulation in hypocotyls; and (v) aldehyde dehydrogenase family 2 member C4-like, which is involved in ferulic acid and sinapic acid biosynthesis by oxidation of coniferyl aldehyde and sinapaldehyde, respectively. In the cell wall, ferulic acid-derived molecules play a major role in cross-linking cell wall-bound polysaccharides to lignins [39].

The second subgroup is related to cell wall structure and contains 14 genes: (i) five genes encode fasciclin-like arabinogalactan protein 12 and 11. These enzymes may affect secondary cell wall composition, architecture, differentiation, elasticity, cell interactions, cell adhesion, and cell wall biosynthesis because they play a role in biomechanics and elastic modulus of the cell wall; (ii) lysine-rich arabinogalactan protein 19-like, which is functionally associated with secondary cell wall thickening and xylem development; (iii) late embryogenesis abundant protein At1g64065-like, a lipid transfer protein that functions as a component of the cuticular lipid export to build the cuticular wax layer; (iv) non-classical arabinogalactan protein 30-like, which contributes to the dynamic remodeling of the cell wall during responses to biotic stresses; (v) protein trichome birefringence-like 23, which is proposed to act as a bridging protein binding pectins and other cell wall polysaccharides and maintaining esterification of pectins; (vi) tubulin beta-1 chain-like, which is associated with cellulose microfibril deposition; (vii) endoglucanase 25 that promotes cell expansion-dependent organ growth; (viii) cellulose synthase A catalytic subunit 7 [UDP-forming], which is a catalytic subunit of cellulose synthase; (ix) ethylene-responsive TF ERF027 that is suggested to function as a master regulator of gene expression during wood formation; and (x) ARGOS-like protein that regulates cell expansion during organ growth (Supplementary Table S6).

These up- and down-regulated genes collectively suggest that overexpression of *PgMyb308-like* in pomegranate hairy roots triggered cell wall reprogramming. In addition, six genes related to the synthesis of shikimate-derived metabolites were downregulated in the *PgMyb308-like-*overexpressing hairy roots. These include: (i) Myb123-like TF that is involved in the biosynthesis of proanthocyanins and anthocyanins; (ii) cytosolic sulfotransferase 5-like that catalyzes the sulfate conjugation of flavones and flavonols; (iii) Myb308 TF that regulates anthocyanin accumulation; (iv) Myb8-like TF that controls the synthesis of phenolamides; (v) bifunctional dihydroflavonol 4-reductase/flavanone 4-reductase that acts in flavonoid metabolism; and (vi) EGL1-like TF that plays a role in activating anthocyanin biosynthesis, probably together with Myb75 (i.e. production of anthocyanin pigment 1/PAP1). The reduction of these genes, particularly the four TFs, may explain the decrease of flavonoids in hairy roots overexpressing *PgMyb308-like*.

### Overexpression of *PgMyb308-like* in *A. thaliana* plants revealed its role in a different genetic and metabolic background

**Figure 6:**
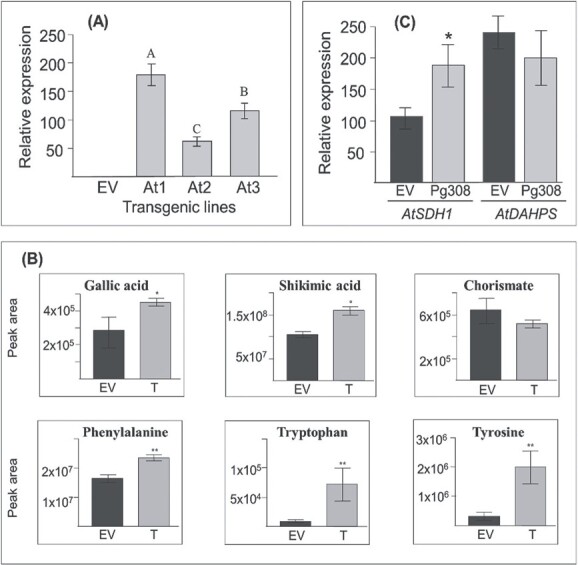
The data obtained from *Arabidopsis thaliana* lines overexpressing *PgMyb308-like.* (A) Relative expression of *PgMyb308-like* in transgenic lines overexpressing *PgMyb308-like* (At1-At3) and control (EV) plants. The expression levels were normalized with the *actin* gene. Each plant line is derived from an independent transformation event. The results are the average of all three transgenic plants, each plant tested in three technical repetitions, with error bars indicating SD. Significantly different (*P* < 0.05) expression, based on the Tukey–Kramer test, was marked by different letters; (B) Levels of gallic acid, shikimate, chorismate and aromatic amino acids (AAAs) in the transgenic lines as determined by GC–MS analysis; (C) Expression analysis of *AtSDH* and *AtDAHPS* in the transgenic lines. Statistical significance was determined based on Student’s *t*-test (*P* < 0.05 and *P* < 0.01) and marked with one and two asterisks, respectively.

To further explore the function of *Pg*Myb308-like in controlling genes in phenolic metabolism, this Myb TF was overexpressed in *A. thaliana* plants that naturally synthesize gallic acid but not HTs. Same plasmid constructs used for pomegranate hairy root transformation were introduced into *Agrobacterium tumefaciens*, which was then used for transformation of *A. thaliana* plants. Twelve independently transformed *A. thaliana* lines were formed together with plants transformed with an EV (i.e. EV lines). Three homozygous transgenic *A. thaliana* lines having the highest *PgMyb308-like* expression were selected for further analysis (Fig. 6A; At1-At3). These lines grew similarly as EV lines (data not shown).

To investigate the metabolic impact of *PgMyb308-like* overexpression, leaves of four-week-old *A. thaliana* plants were analyzed using GC–MS. As with the transgenic pomegranate hairy roots, shikimate, tyrosine, tryptophan, and phenylalanine were increased by 54%, 7.8-fold, 60%, and 7.7-fold, respectively, in the *A. thaliana* lines that overexpressed *PgMyb308-like* (Fig. 6B). However, unlike what was observed in hairy roots, gallic acid was increased by 60% in *PgMyb308-lik*e-overexpressing *A. thaliana* lines (Fig. 6B).

To understand the underlying mechanisms for the higher levels of AAAs, the expression of *AtSDH* and *AtDAHPS* was determined by quantitative real-time PCR. *A. thaliana* has a single gene encoding *At*SDH (with higher sequence similarity to *Vv*SDH1 and *Pg*SDH1 than the other *V. vinifera* and pomegranate SDH isoforms), unlike the multiple SDH isozymes found in *V. vinifera* and pomegranate [20, 30]. *AtSDH* was upregulated by 65% in *PgMyb308-like-*overexpressing *A. thaliana* lines relative to the EV lines*,* unlike *AtDAHPS* with a non-significantly changed expression (Fig. 6C).

As revealed by the LC–MS/MS analysis, the levels of three *p*-hydroxybenzoic acid derivatives: 4-hydroxybenzoic acid, 3,4-dihydroxybenzoic acid, and orbicularin (2,5-dihydroxybenzoic acid 2-*O*-glucopyranoside), were elevated in the *PgMyb308-like-*overexpressing *A. thaliana* lines compared to the EV control lines (Supplementary Table S7). Additionally, quercetin, coniferol, pelargonidin, cyanidin 3-D-rutinoside betaine, cyanidin 3,5-diglucoside, and pelargonidin 3-*O*-rutinoside betaine were significantly increased. By contrast, the levels of chorismate, *p*-coumaric acid, coumarin, isoflavone-7-glucoside, isoquercetin, and leucodelphinidin were significantly reduced (Supplementary Table S7). The PCA analysis of the LC–MS/MS data also showed that the three lines overexpressing the *PgMyb308-like* varied from the EV lines for their metabolite profiles (Supplementary Fig. S5).

These results showed that similar to the *PgMyb308-like*-overexpressing pomegranate hairy roots, *Pg*Myb308-like can also regulate *AtSDH* expression and the accumulation of metabolites in the shikimate pathway in *A. thaliana* as a high level of shikimate and AAAs were detected. However, it does appear to affect different metabolites downstream of chorismate and AAAs than those in the pomegranate hairy roots overexpressed *PgMyb308-like*. These differential impacts on metabolite profiles could be due to (i) the different genetic backgrounds (pomegranate vs. *A. thaliana*); (ii) the fact that pomegranate is a perennial plant and *A. thaliana* is an annual plant; (iii) the differences in the metabolic pathways and their regulations, for example *A. thaliana* forms gallic acid but does not produce HTs; and (iv) the different tissue types being analyzed (hairy roots of pomegranate vs. rosettes leaves of *A. thaliana*).

## Conclusion

In this study, we isolated and characterized a new R2R3-Myb TF, *Pg*Myb308-like, from the outer peels of pomegranate fruit. When overexpressed in pomegranate hairy roots, *Pg*Myb308-like led to lower levels of gallic acid and most HTs, but higher accumulation of shikimate and its downstream metabolites AAAs, apparently through regulating different isoforms of the branching point gene *PgSDH*. *Pg*Myb308-like also suppressed AAA-derived phenolic metabolites such as flavonoids, but increased the level of total lignins. The transcriptome analysis further revealed reprogramming of cell wall related genes upon overexpression of *PgMyb308-like* in pomegranate hairy roots. Further gene expression and metabolite analyses of *PgMyb308-like-*overexpresssing *A. thaliana* plants suggested that *Pg*Myb308-like functions similarly with regard to the control of *SDH* expression and the levels of shikimate and AAAs, but distinctly towards flavonoids and other specialized metabolites in *A. thaliana*. This might occur due to the different genetic and metabolic context, pathway regulation, and tissue types in *A. thaliana* than pomegranate. Overall, our integrated transcriptome and metabolite analysis provides new insights into the function of *Pg*Myb308-like in modulating the production and accumulation of a wide range of metabolites that play key roles in pomegranate physiology and nutrient content. This knowledge could be used to enhance the nutritional and commercial quality of pomegranate.

## Materials and methods

### Generation of pomegranate and Arabidopsis plants overexpressing *PgMyb308-like*

Total RNA was extracted from the outer fruit peels of pomegranate accession “Wonderful” using a Cetyltrimethylammonium bromide (CTAB)-based method. One μg of total RNA used to form cDNA by using the PrimeScript™ RT-PCR kit with gDNA Eraser (Clontech, Fremont, CA).

The open reading frame of *PgMyb308-like* was amplified using primers listed in Supplementary Table S8, and cloned into the plasmid vector pENTR/D-TOPO and then to pK7WG2D (Invitrogen, Carlsbad, CA). The pK7WG2D vector contains the *neomycin phosphotransferase* (*nptII*), the enhanced green fluorescent protein-endoplasmic reticulum (*EGFP-ER*) reporter genes, and the *cauliflower mosaic virus 35S* (*CaMV 35S*) promoter. The binary vector with (i.e. overexpression) or without (i.e. empty vector control) *PgMyb308-like* was transformed into *A. rhizogenes* strain MSU440 by electroporation (Bio-Rad; conditions: 1.8 kV, 25 μF, 200 Ω). For generating transgenic pomegranate hairy roots, two-week-old pomegranate seedlings cv. PG116–17 were used for inducing hairy roots after cutting a small section of their roots tip. Hairy root induction and propagation were performed as previously described (Ono et al. 2016). Two weeks after co-cultivation with *A. rhizogenes*, the infected roots began to form hairy roots. Each of the GFP-positive hairy root induced from each seedling, was used as a replicate.

The same plasmid constructs used for *A. rhizogenes* transformation was also transformed into *A. tumefaciens* strain GV3101. *A. thaliana* plants were transformed using the floral dip method [40]. Selection of transformed seeds was carried out on an agar medium supplemented with kanamycin (50 mg mL^−1^; Duchefa Biochemie B.V., Haarlem, the Netherlands). Fifteen independent transformation events were selected, and T_3_ homozygous lines were obtained as described previously [41]. As a control, plants expressing an empty vector were generated using the same method described above (designated as EV).

### Multiple sequence alignments and phylogenetic analysis

Multiple sequence alignments were performed using *T-Coffee* with a gap open penalty of 10 and a gap extension penalty of 0.2 [42]. The multiple sequence alignment file was shaded using the BoxShade program (https://embnet.vital-it.ch/software/BOX_form.html). Phylogenetic analysis was carried out in MEGA5 (https://www.megasoftware.net/) using selected Myb TF protein sequences (see accession numbers in the Figure legend).

### GC–MS analysis

Transgenic pomegranate hairy roots and *A. thaliana* leaves were frozen in liquid nitrogen, and then dried with a lyophilizer. The lyophilized tissues were grounded into a fine powder with a mortar and pestle. Determination of amino acids, chorismate, gallic acid, and shikimate was conducted using GC–MS analysis [41].

### LC–MS/MS analysis

Twenty mg of the hairy root powder and 50 mg of ground *A. thaliana* leaves were placed in 2-ml tubes, and 1 ml of extraction solvent (80% methanol, 20% double distilled water, and 0.1% HCl) was added. The tubes were incubated in the dark at room temperature on a shaker for 1 h. Following centrifugation at 20817 x g at 4°C for 20 min, the supernatant was collected and filtered by a syringe filter (0.22 μm) into HPLC vials and placed in −20°C for storage until analysis. Two $ \square$ $l of the extract was injected into an uHPLC connected to a photodiode array detector (Dionex Ultimate 3000) with a reverse-phase column (Phenomenex C18-column, Synergi 2.5 $ \square$ $m, Hydro-RP, 100 A° 100 mm × 3 mm). The mobile phase comprised of (A) double distilled water with 0.1% formic acid and (B) acetonitrile containing 0.1% formic acid. The gradient started with 5% B and increased to 90% B in 15 min, then increased to 98% B in 1 min, and kept isocratic at 98% B for 2 min. Phase B returned to 5% in 2 min, and the column was allowed to equilibrate at 5% B for 2 min before the next injection. The flow rate was maintained at 0.4 ml min^−1^.

The LC–MS/MS analysis was performed as we previously described [43]. For the MS/MS analysis, the collision energy was set to 15, 50, and 100 electron volt (eV).

Peak determination and peak area integration were performed with Compound Discoverer 3.1 (Thermo Xcalibur, Version 3.1.0.305) and QuanBrowser (Thermo Xcalibur, Version 4.1.31.9). For some of the compounds, identification was performed based on comparison to the authentic standards. Others were recognized based on the MzCloud database using the ChemSpider database and high-resolution mass spectrometry (HRMS) data.

### Analysis of total lignin content

Lignin analysis of pomegranate hairy roots was done as described [44, 45] with minor modifications. Hairy roots were collected, lyophilized, and ground into fine powder. Ten mg of hairy root powder was extracted with 0.5 ml of 50% ethanol at 80°C. After 3 h, 0.5 ml of methanol was added and then incubated for an additional 1 h at 80°C. The samples were centrifuged at 20000 x g for 20 min. The supernatant was discarded, and the remaining residue was dried at 60°C. The remaining steps of the process were performed as described previously [45].

### RNA extraction and real-time quantitative PCR analysis

Total RNA was extracted from the three pomegranate transgenic hairy root lines overexpressing *PgMyb308-like*, and from three lines of EV control. For the RNA extraction, 100 mg of fresh hairy root samples were extracted using the Spectrum plant total RNA kit (Sigma-Aldrich). Total RNA was treated with RNase-free DNase I (Sigma-Aldrich). cDNA was synthesized using the High Capacity cDNA Reverse Transcription Kit (Applied Biosystem, Inc, Foster City, CA, USA). The housekeeping *ribosomal Protein S* (*PgRPSII*) gene was used to normalize variance among hairy root samples. For analysis with *A. thaliana* samples, the housekeeping *AtPP2A* gene was used for normalization [46]. Means of three biological replicates, each with three technical replicates, were used for the gene expression analysis. The primers used in the real time quantitative PCR analysis are provided in Supplementary Table S7.

### Library construction, sequencing, and sequence data analysis

The total RNA for each sample was processed using the TranSeq protocol and the overall workflow was as previously described (Tzfadia et al., 2018). Quality of the libraries was evaluated by Qubit (Thermo fisher scientific) and TapeStation (Agilent). Sequencing libraries were constructed as previously described [47]. Reads for each sample were aligned independently to the pomegranate (*P. granatum* L.) reference genome GCA_007655135.2 using STAR (2.4.2a) [45], and the annotations based on the NCBI. TranSeq analysis data were also mapped using Bowtie2 v2.2.5 to the pomegranate genome [46]. The percentage of the reads that were aligned uniquely to the genome was 79%. Expression levels for each gene were quantified using htseq-count (version 0.6.1p1). Reads were counted on the last bases of the gene, and extend the 3′-untranslated region (UTR) exon downstream the transcript (3’-UTR based gtf). Differential analysis was performed as described [48].

### GO analysis

To identify the GO terminologies for all statistically significantly expressed genes, Blast2Go software (www.blast2go.com) was used [48]. GO enrichment analysis on the DEGs was performed using the PANTHER Overrepresentation test [48] available at the GO web tool (http://geneontology.org/). The default parameter settings were used for the enrichment analysis [i.e. Fisher’s exact **test**, after false discovery rate (FDR) correction], with the *A. thaliana* genome used as a reference list. BP and CC GO terms were considered significantly enriched if they had an FDR corrected *P value* ≤ 0.05.

## Statistical analysis

Statistical analysis was carried out using JMP software version 8.0 (SAS Institute Inc., Cary, NC). Significant differences between groups were calculated according to the Turkey-Kramer HSD test (*P* ≤ 0.05).

## Acknowledgments

The study was supported by the US-Israel Binational Agricultural Research and Development Fund (BARD) project numbers IS-4822-15R and IS-5403-21CR, as well as the Migal Research Institute.

## Author contribution

RD, UY, LT and RA conducted the study; LT and RA initiated the study; RD and UY carried out the experiments and the analysis; RA and LT wrote the manuscript. All authors have read and approved the manuscript.

## Data availability

All data supporting the findings of this study are available within the paper and within its supplementary materials published online. The FASTQ data is available at GEO accession GSE178072.

## Conflict of Interest statement

The authors declare that they have no competing and conflict interests.

## Supplementary data

Supplementary data is available at *Horticulture Research Journal* online.

## Supplementary Material

Web_Material_uhab008
