# Supplementary material for: A Myb transcription factor, PgMyb308-like, enhances the level of shikimate, aromatic amino acids, and lignins, but represses the synthesis of flavonoids and hydrolyzable tannins, in pomegranate (Punica granatum L.)
Source: Hortic Res. 2022 Feb 11;9:uhac008. doi: 10.1093/hr/uhac008 (PMC9113223; doi:10.1093/hr/uhac008)
Supplement: Web_Material_uhab008 [file Web_Material_uhab008.zip › Web_Material_uhab008.pdf]

Supplementary Figures

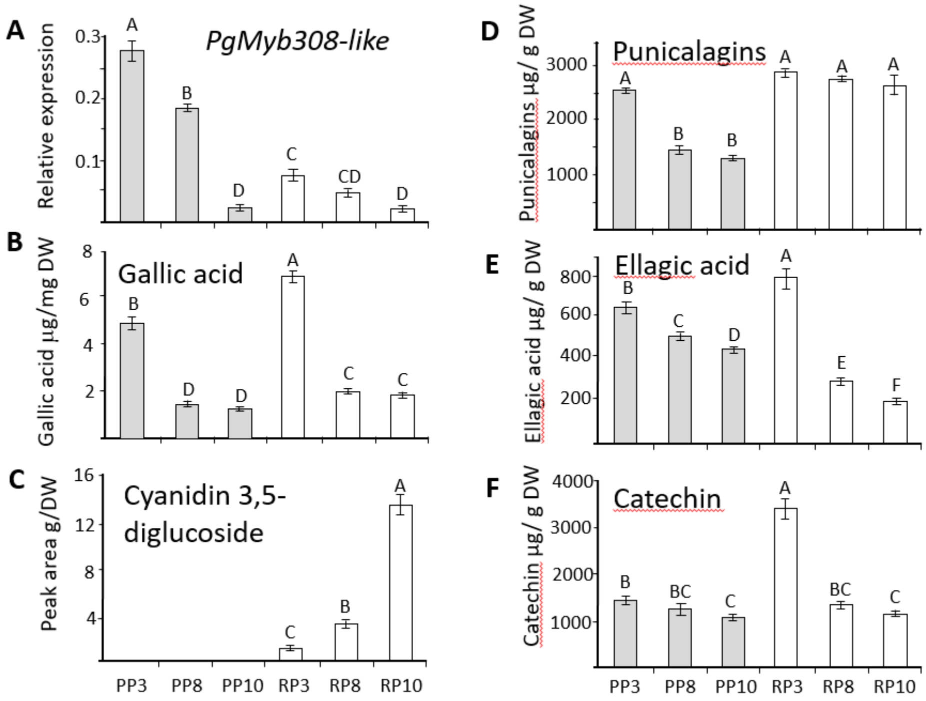

Supplementary Figure S1. The expression level of *PgMyb308-like* (A), and the levels of several shikimate-related metabolites (B-F), in the outer peels of two accessions, PG200-211 (pink peel, PP) and PG116-17 (red peel, RP). The samples were taken from three fruit developmental stages [young fruit (stages 3), nearly mature fruit (stage 8), and ripened fruit (stage 10) (according to references [21,22])]. Expression of *PgMyb308-like* has normalized with the pomegranate *Ribosomal Protein S* gene. The concentrations of gallic acid and cyanidin 3,5-diglucoside were analyzed using HPLC, while punicalagins, ellagic acid, and catechin were determined using LC-MS/MS. At each developmental stage, fruits were sampled from three trees of each cultivar. Three fruits from each tree were pooled together for the *PgMyb308-like* expression analysis (each tree serving as a biological replicate). Therefore, the results are average ± SE of three biological repeats. However, for the metabolite determination, each fruit was used as a biological repeat; thus, the results are average ± SE of nine biological repeats. Different letters indicate significant differences ($P < 0.05$), based on the Tukey-Kramer test.

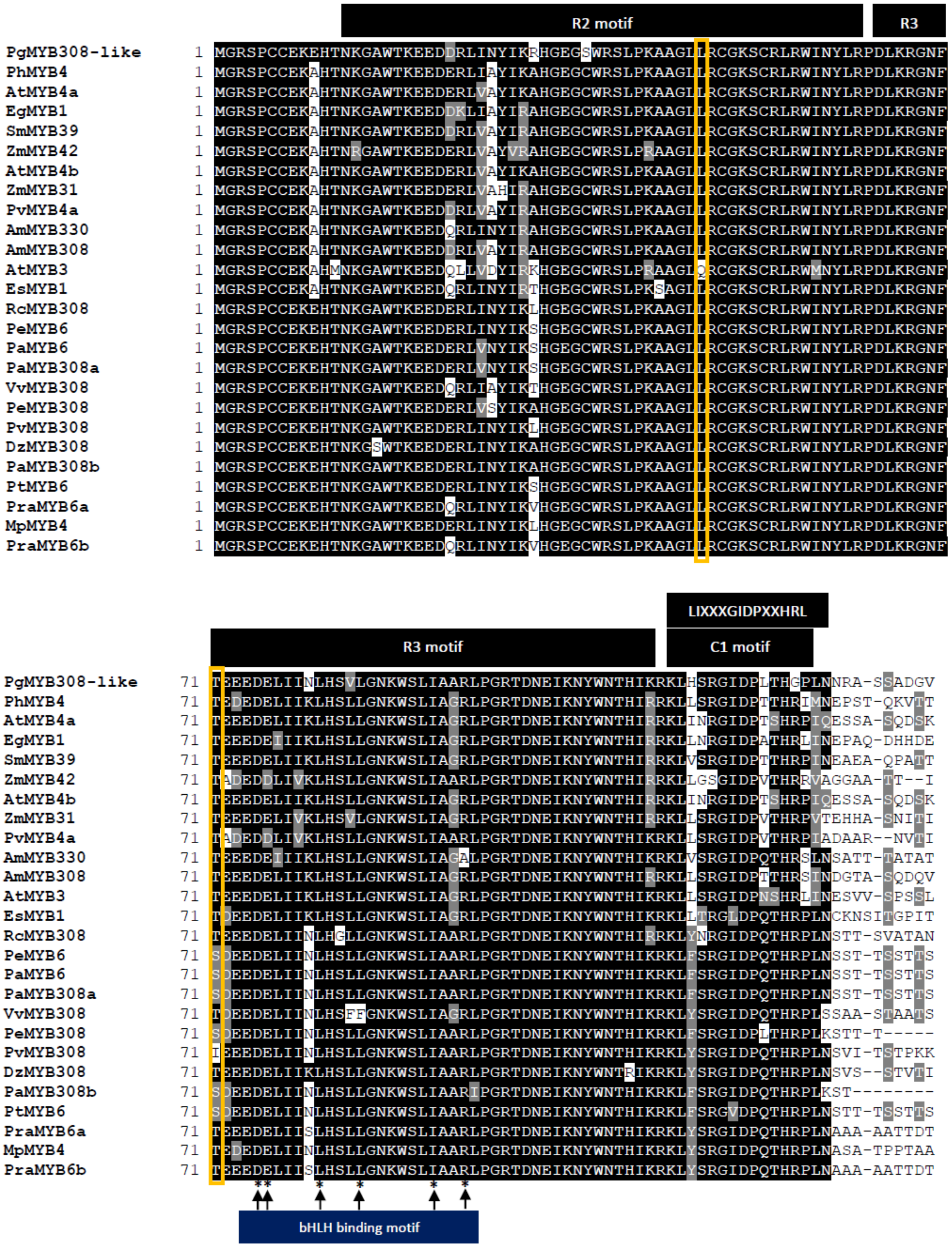
R2 motif
R3
PgMYB308-like 1 MGRSPCCEKEHTNKGAWTKEEDDRLINYIKRHGEGSWRSLPKAAGLLRCGKSCRLRWINYLRPDLKRGNF
PhMYB4 1 MGRSPCCEKAHTNKGAWTKEEDERLIAYIKAHGEGCWRSLPKAAGLLRCGKSCRLRWINYLRPDLKRGNF
AtMYB4a 1 MGRSPCCEKAHTNKGAWTKEEDERLVAYIKAHGEGCWRSLPKAAGLLRCGKSCRLRWINYLRPDLKRGNF
EgMYB1 1 MGRSPCCEKAHTNKGAWTKEEDDKLIAYIRAHGEGCWRSLPKAAGLLRCGKSCRLRWINYLRPDLKRGNF
SmMYB39 1 MGRSPCCEKAHTNKGAWTKEEDDRLVAYIRAHGEGCWRSLPKAAGLLRCGKSCRLRWINYLRPDLKRGNF
ZmMYB42 1 MGRSPCCEKAHTNRGAWTKEEDERLVAYVRAHGEGCWRSLPRAAGLLRCGKSCRLRWINYLRPDLKRGNF
AtMYB4b 1 MGRSPCCEKAHTNKGAWTKEEDERLVAYIKAHGEGCWRSLPKAAGLLRCGKSCRLRWINYLRPDLKRGNF
ZmMYB31 1 MGRSPCCEKAHTNKGAWTKEEDERLVAHIRAHGEGCWRSLPKAAGLLRCGKSCRLRWINYLRPDLKRGNF
PvMYB4a 1 MGRSPCCEKAHTNKGAWTKEEDDRLVAYIRAHGEGCWRSLPKAAGLLRCGKSCRLRWINYLRPDLKRGNF
AmMYB330 1 MGRSPCCEKAHTNKGAWTKEEDQRLINYIRAHGEGCWRSLPKAAGLLRCGKSCRLRWINYLRPDLKRGNF
AmMYB308 1 MGRSPCCEKAHTNKGAWTKEEDDRLVAYIRAHGEGCWRSLPKAAGLLRCGKSCRLRWINYLRPDLKRGNF
AtMYB3 1 MGRSPCCEKAHMNKGAWTKEEDQLLVDYIRKHGEGCWRSLPRAAGLQRCGKSCRLRWMNYLRPDLKRGNF
EsMYB1 1 MGRSPCCEKAHTNKGAWTKEEDQRLINYIRTHGEGCWRSLPKSAGLLRCGKSCRLRWINYLRPDLKRGNF
RcMYB308 1 MGRSPCCEKEHTNKGAWTKEEDERLINYIKLHGEGCWRSLPKAAGLLRCGKSCRLRWINYLRPDLKRGNF
PeMYB6 1 MGRSPCCEKEHTNKGAWTKEEDERLINYIKSHGEGCWRSLPKAAGLLRCGKSCRLRWINYLRPDLKRGNF
PaMYB6 1 MGRSPCCEKEHTNKGAWTKEEDERLVNYIKSHGEGCWRSLPKAAGLLRCGKSCRLRWINYLRPDLKRGNF
PaMYB308a 1 MGRSPCCEKEHTNKGAWTKEEDERLVNYIKSHGEGCWRSLPKAAGLLRCGKSCRLRWINYLRPDLKRGNF
VvMYB308 1 MGRSPCCEKEHTNKGAWTKEEDQRLIAYIKTHGEGCWRSLPKAAGLLRCGKSCRLRWINYLRPDLKRGNF
PeMYB308 1 MGRSPCCEKEHTNKGAWTKEEDERLVSYIKAHGEGCWRSLPKAAGLLRCGKSCRLRWINYLRPDLKRGNF
PvMYB308 1 MGRSPCCEKEHTNKGAWTKEEDERLINYIKLHGEGCWRSLPKAAGLLRCGKSCRLRWINYLRPDLKRGNF
DzMYB308 1 MGRSPCCEKEHTNKGSWTKEEDERLINYIKAHGEGCWRSLPKAAGLLRCGKSCRLRWINYLRPDLKRGNF
PaMYB308b 1 MGRSPCCEKEHTNKGAWTKEEDERLINYIKAHGEGCWRSLPKAAGLLRCGKSCRLRWINYLRPDLKRGNF
PtMYB6 1 MGRSPCCEKEHTNKGAWTKEEDERLINYIKSHGEGCWRSLPKAAGLLRCGKSCRLRWINYLRPDLKRGNF
PraMYB6a 1 MGRSPCCEKEHTNKGAWTKEEDQRLINYIKVHGEGCWRSLPKAAGLLRCGKSCRLRWINYLRPDLKRGNF
MpMYB4 1 MGRSPCCEKEHTNKGAWTKEEDERLINYIKLHGEGCWRSLPKAAGLLRCGKSCRLRWINYLRPDLKRGNF
PraMYB6b 1 MGRSPCCEKEHTNKGAWTKEEDQRLINYIKVHGEGCWRSLPKAAGLLRCGKSCRLRWINYLRPDLKRGNF
LIXXXGIDPXXHRL
R3 motif
C1 motif
PgMYB308-like 71 TEEEDELIINLHSVLGNKWSLIAARLPGRTDNEIKNYWNTHIKRKLHSRGIDPLTHGPLNNRA-SSADGV
PhMYB4 71 TEDEDELIIKLHSLLGNKWSLIAGRLPGRTDNEIKNYWNTHIRRKLLSRGIDPTTHRIMNEPST-QKVTT
AtMYB4a 71 TEEEDELIIKLHSLLGNKWSLIAGRLPGRTDNEIKNYWNTHIRRKLINRGIDPTSHRPIQESSA-SQDSK
EgMYB1 71 TEEEDEIIIKLHSLLGNKWSLIAGRLPGRTDNEIKNYWNTHIRRKLLNRGIDPATHRLINEPAQ-DHHDE
SmMYB39 71 TEEEDELIIKLHSLLGNKWSLIAGRLPGRTDNEIKNYWNTHIRRKLVSRGIDPTTHRPINEAEA-QPATT
ZmMYB42 71 TADEDDLIVKLHSLLGNKWSLIAARLPGRTDNEIKNYWNTHIRRKLLGSGIDPVTHRRVAGGAA-TT--I
AtMYB4b 71 TEEEDELIIKLHSLLGNKWSLIAGRLPGRTDNEIKNYWNTHIRRKLINRGIDPTSHRPIQESSA-SQDSK
ZmMYB31 71 TEEEDELIVKLHSVLGNKWSLIAGRLPGRTDNEIKNYWNTHIRRKLLSRGIDPVTHRPVTEHHA-SNITI
PvMYB4a 71 TADEDDLIVKLHSLLGNKWSLIAARLPGRTDNEIKNYWNTHIKRKLLSRGIDPVTHRPIADAAR--NVTI
AmMYB330 71 TEEEDEIIIKLHSLLGNKWSLIAGALPGRTDNEIKNYWNTHIKRKLVSRGIDPQTHRSLNSATT-TATAT
AmMYB308 71 TEEEDELIIKLHSLLGNKWSLIAGRLPGRTDNEIKNYWNTHIRRKLLSRGIDPTTHRSINDGTA-SQDQV
AtMYB3 71 TEEEDELIIKLHSLLGNKWSLIAGRLPGRTDNEIKNYWNTHIKRKLLSRGIDPNSHRLINESVV-SPSSL
EsMYB1 71 TDEEDELIIKLHSLLGNKWSLIAGRLPGRTDNEIKNYWNTHIKRKLLTRGLDPQTHRPLNCKNSITGPIT
RcMYB308 71 TEEEDELIINLHGLLGNKWSLIAARLPGRTDNEIKNYWNTHIRRKLYNRGIDPQTHRPLNSTT-SVATAN
PeMYB6 71 SDEEDELIINLHSLLGNKWSLIAARLPGRTDNEIKNYWNTHIKRKLFSRGIDPQTHRPLNSST-TSSTTS
PaMYB6 71 SDEEDELIINLHSLLGNKWSLIAARLPGRTDNEIKNYWNTHIKRKLFSRGIDPQTHRPLNSST-TSSTTS
PaMYB308a 71 SDEEDELIINLHSLLGNKWSLIAARLPGRTDNEIKNYWNTHIKRKLFSRGIDPQTHRPLNSST-TSSTTS
VvMYB308 71 TDEEDELIINLHSFFGNKWSLIAGRLPGRTDNEIKNYWNTHIKRKLYSRGIDPQTHRPLSSAA-STAATS
PeMYB308 71 SDEEDELIINLHSLLGNKWSLIAARLPGRTDNEIKNYWNTHIKRKLFSRGIDPLTHRPLKSTT-T-----
PvMYB308 71 IEEEDELIINLHSLLGNKWSLIAARLPGRTDNEIKNYWNTHIKRKLYSRGIDPQTHRPLNSVI-TSTPKK
DzMYB308 71 TEEEDELIIKLHSLLGNKWSLIAARLPGRTDNEIKNYWNTRIKRKLYSRGIDPQTHRPLNSVS--STVTI
PaMYB308b 71 SDEEDELIINLHSLLGNKWSLIAARIPGRTDNEIKNYWNTHIKRKLFSRGIDPQTHRPLKST--------
PtMYB6 71 SDEEDELIINLHSLLGNKWSLIAARLPGRTDNEIKNYWNTHIKRKLFSRGVDPQTHRPLNSTT-TSSTTS
PraMYB6a 71 TEEEDELIISLHSLLGNKWSLIAARLPGRTDNEIKNYWNTHIKRKLYSRGIDPQTHRPLNAAA-AATTDT
MpMYB4 71 TEDEDELIINLHSLLGNKWSLIAARLPGRTDNEIKNYWNTHIKRKLYSRGIDPQTHRPLNASA-TPPTAA
PraMYB6b 71 TEEEDELIISLHSLLGNKWSLIAARLPGRTDNEIKNYWNTHIKRKLYSRGIDPQTHRPLNAAA-AATTDT
* * * * * *
bHLH binding motif

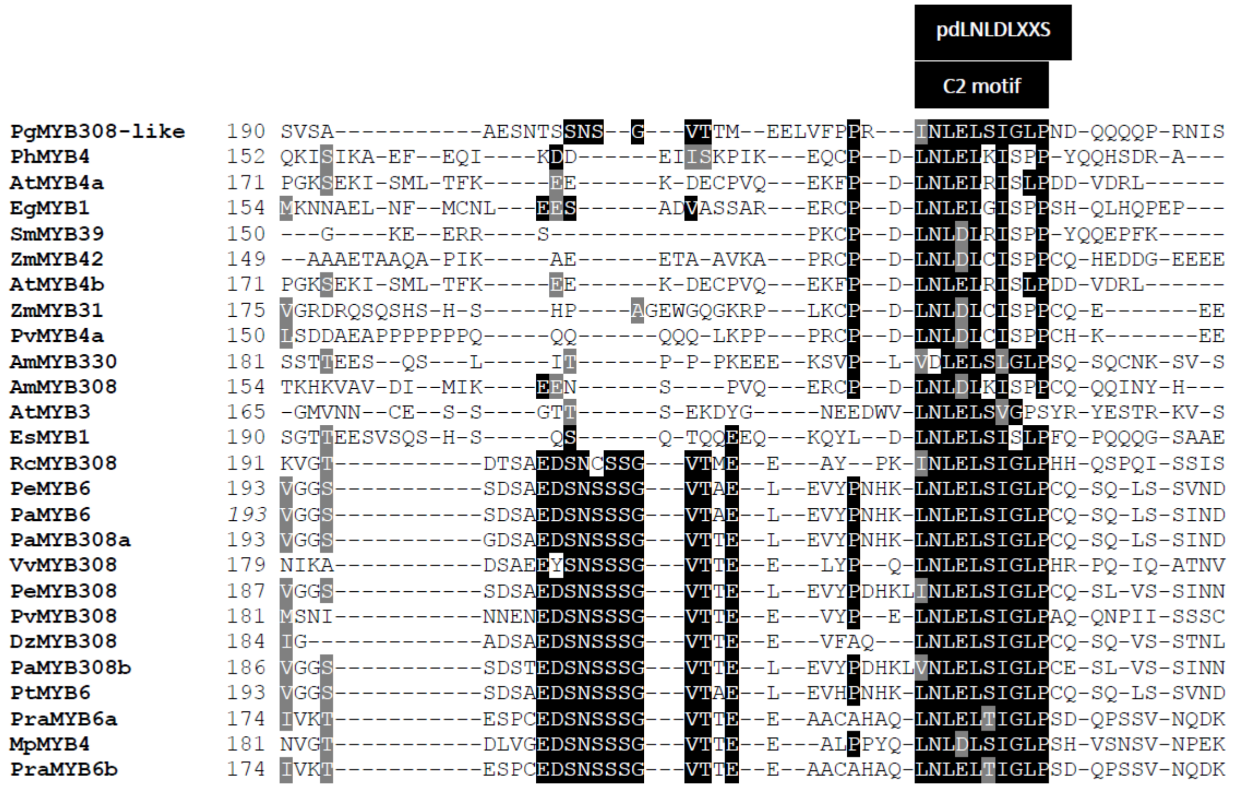

Supplementary Figure S2. Sequence analysis of the pomegranate *Pg*Myb308-like transcription factor. Alignment of the amino acid sequences of *Pg*Myb308-like and other R2R3-Myb subgroup S4 proteins. The conserved domains and motifs are shown. Black letters on a white background represent amino acids that are not conserved, while white letters on a black background represent residues that are identical in the sequences aligned. White letters on a gray background indicate conservative changes. Boxes indicate conserved elements in the primary sequence, including the R2 and R3 Myb domains; the C1 motif, LLsrGIDPX(T/S)HRX(I/L); the C2 motif, pdLNL(D/E)LXi(G/S); and the bHLH-binding region. The sequences used for this analysis and their GenBank accession numbers are: *Punica granatum* ***PgMYB308-like***, AIO09658; *Petunia x hybrida* ***PhMYB4***, ADX33331.1; *Arabidopsis thaliana* ***AtMYB4a***, AAS10085.1; *Eucalyptus gunnii* ***EgMYB1***, CAE09058.1; *Salvia miltiorrhiza* ***SmMYB39***, AGS55356.1; *Zea mays* ***ZmMYB42***, NP_001106009.2; *Arabidopsis thaliana* ***AtMYB4b***, NP_195574.1; *Zea mays* ***ZmMYB31***, CAJ42202.1; *Panicum virgatum* ***PvMYB4a***, AEM17348.1; *Antirrhinum majus* ***AmMYB330***, P81395.1; *Antirrhinum majus* ***AmMYB308***, P81393.1; *Arabidopsis thaliana*, ***AtMYB3***, NP_564176.2; *Epimedium sagittatum* ***EsMYB1***, AFH03053.1; *Ricinus communis* ***RcMYB308***, XP_025012074.1; *Populus euphratica* ***PeMYB6***, XP_011001250.1; *Populus alba* ***PaMYB6***, XP_034893483.1; *Populus alba* ***PaMYB308a***, XP_034918624.1; *Vitis vinifera* ***VvMYB308***, XP_019076420.1; *Populus euphratica* ***PeMYB308***, XP_011007997.1; *Pistacia vera* ***PvMYB308***, XP_031286938.1; *Durio zibethinus* ***DzMYB308***, XP_022773827.1; *Populus alba* ***PaMYB308b***, XP_034917141.1; *Populus trichocarpa* ***PtMYB6***, XP_002325733.1; *Prosopis alba* ***PraMYB6a***, XP_028766124.1; *Mucuna pruriens* ***MpMYB4***, RDX63213.1; *Prosopis alba* ***PraMYB6b***, XP_028785141.1.

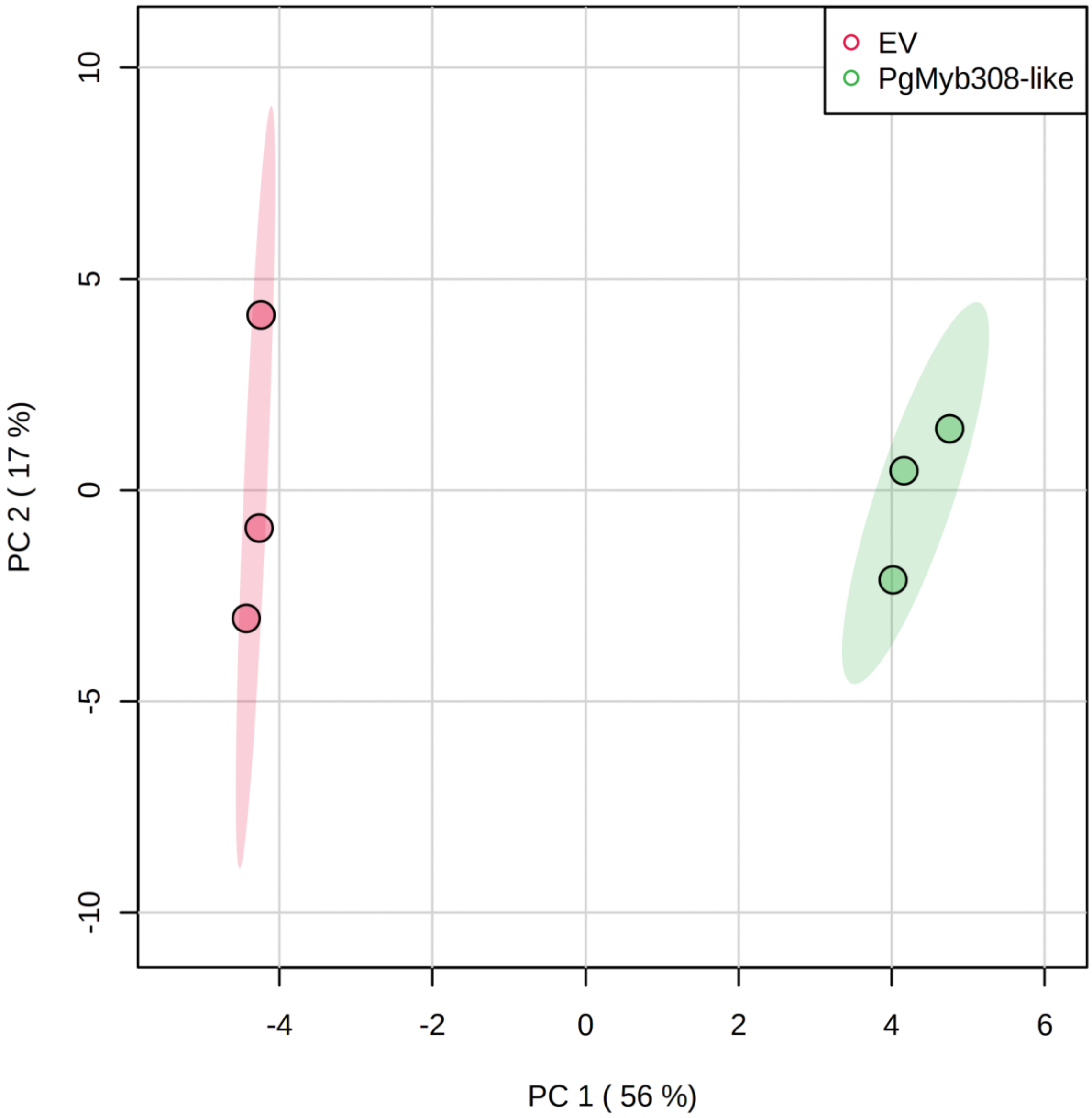

Supplementary Figure S3. Principal component analysis of LC-MS metabolite profiling data of pomegranate hairy roots overexpressing *PgMyb308-like* or those transformed with an empty vector (EV). Variance explained by each component is indicated in parenthesis.

(A)

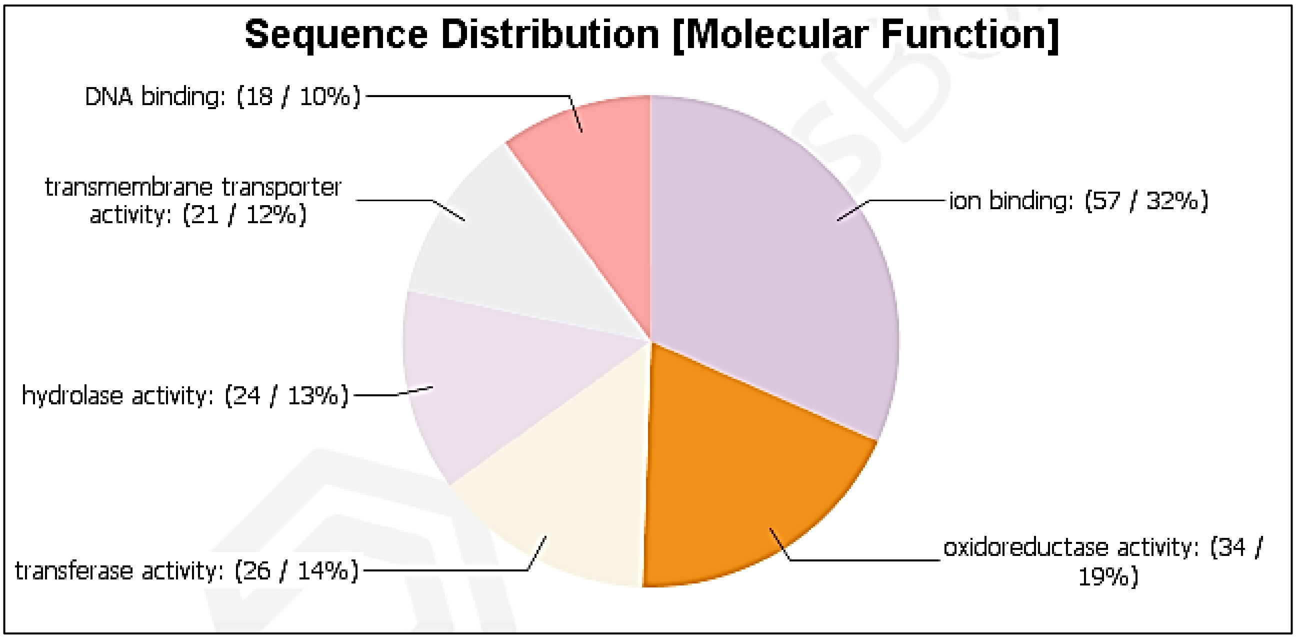

(B)

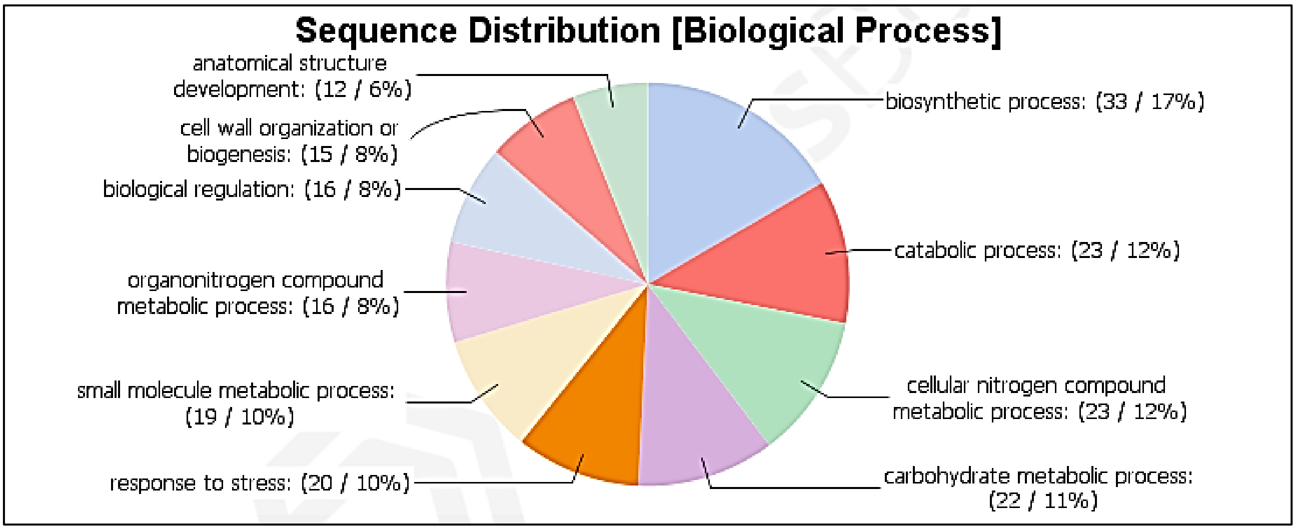

Supplementary Figure S4. Gene ontology analysis of differentially expressed genes in pomegranate hairy roots overexpressing *PgMyb308-like* and those transformed with an empty vector (EV). Pie charts of Gene Ontology categories of (A) molecular function, and (B) biological process are shown. Note that any one probe can belong to more than one Gene Ontology category.

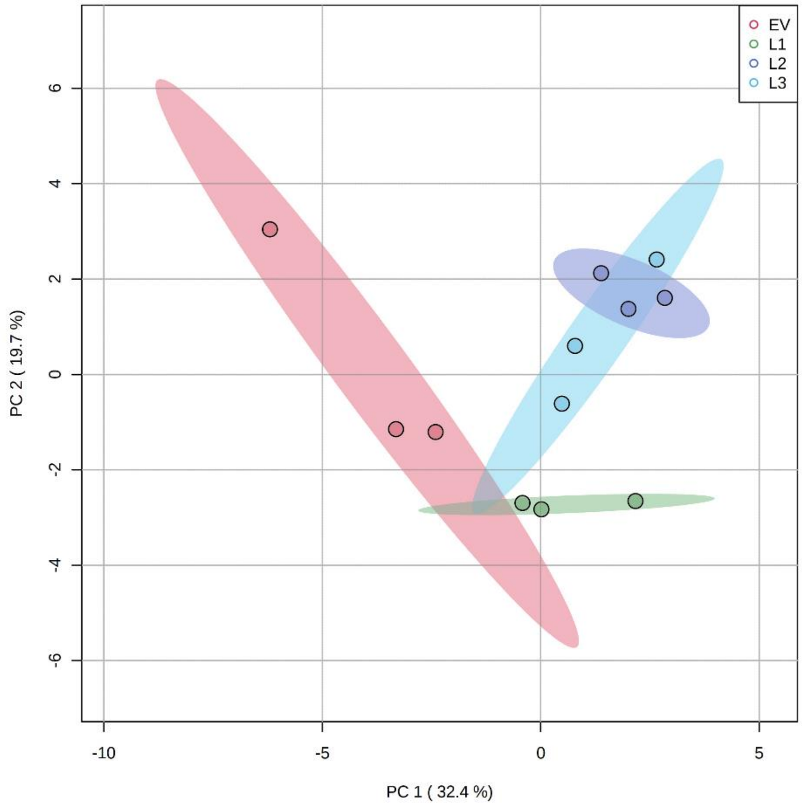

Supplementary Figure S5. Principal component analysis of LC-MS/MS metabolite profiling data of *PgMyb308-like-overexpressing Arabidopsis thaliana* plants and those transformed with an empty vector (EV). Variance explained by each component is indicated in brackets.
